# Non-adherence to ivermectin in onchocerciasis-endemic communities with persistent infection in the Bono Region of Ghana: a mixed-methods study

**DOI:** 10.1186/s12879-023-08806-8

**Published:** 2023-11-16

**Authors:** Kenneth Bentum Otabil, María-Gloria Basáñez, Blessing Ankrah, Emmanuel John Bart-Plange, Theophilus Nti Babae, Prince-Charles Kudzordzi, Vera Achiaa Darko, Abdul Sakibu Raji, Lydia Datsa, Andrews Agyapong Boakye, Michael Tawiah Yeboah, Joseph Nelson Siewe Fodjo, Henk D. F. H. Schallig, Robert Colebunders

**Affiliations:** 1 Centre for Research in Applied Biology, School of Sciences, University of Energy and Natural Resources, Sunyani, Bono Region Ghana; 2 Department of Biological Science, School of Sciences, University of Energy and Natural Resources, Sunyani, Bono Region Ghana; 3 Global Health Institute, Faculty of Medicine and Health Sciences, University of Antwerp, Antwerp, Belgium; 4 Department of Infectious Disease Epidemiology, MRC Centre for Global Infectious Disease Analysis (MRC GIDA), London Centre for Neglected Tropical Disease Research, School of Public Health, Imperial College London, London, UK; 5 STU Clinic, Sunyani Technical University, Sunyani, Bono Region Ghana; 6 Deo Gratias Medical Laboratories, Sunyani, Bono Region Ghana; 7 Kintampo Health Research Centre, Kintampo, Bono Region Ghana; 8 Ghana Health Service, Regional Neglected Tropical Diseases (RNTD) Office, Regional Health Directorate, Sunyani, Bono Region Ghana; 9 Department of Medical Microbiology, Experimental Parasitology Unit, Academic Medical Centre at the University of Amsterdam, Amsterdam University Medical Centres, Amsterdam, The Netherlands

**Keywords:** Onchocerciasis, Ivermectin, Mass drug administration, Non-adherence, Mixed-methods, Ghana

## Abstract

**Background:**

The World Health Organization has proposed that onchocerciasis elimination (interruption) of transmission be verified in 12 (approximately a third) endemic countries by 2030. The strategy to reach this goal is based on ivermectin Mass Drug Administration (MDA) with high geographical and therapeutic coverage. In addition to coverage, high levels of treatment adherence are paramount. We investigated factors associated with ivermectin intake in an area of Ghana with persistent *Onchocerca volvulus* infection.

**Methods:**

In August 2021, a cross-sectional mixed-methods study was conducted in 13 onchocerciasis-endemic communities in the Bono Region of Ghana. Individuals aged ≥ 10 years were invited to participate in a questionnaire survey. A total of 48 focus group discussions and in-depth interviews with 10 community drug distributors and 13 community leaders were conducted.

**Results:**

A total of 510 people participated in the study [median age: 32, interquartile range 30 (20‒50) years]; 274 (53.7%) were females. Of the total, 320 (62.7%) declared that they adhered to each treatment round and 190 (37.3%) admitted they had not taken ivermectin during at least one MDA round, since becoming eligible for treatment. Of 483 participants with complete information, 139 (28.8%) did not take ivermectin during the last round (March 2021), and 24 (5.0%) had never taken ivermectin (systematic non-adherers). Reasons for not taking ivermectin included previous experience/fear of side-effects, being absent during MDA, pregnancy, the desire to drink alcohol, and drug distribution challenges. Being male, having good knowledge and perception of the disease, and not having secondary or higher level of formal education were significantly associated with higher odds of ivermectin intake.

**Conclusions:**

A relatively high level of non-adherence to ivermectin treatment was documented. There is a need for targeted educational and behavioural change campaigns to reverse these trends and ensure a steady course toward meeting onchocerciasis elimination targets in Ghana.

**Supplementary Information:**

The online version contains supplementary material available at 10.1186/s12879-023-08806-8.

## Introduction

Onchocerciasis is a debilitating neglected tropical disease (NTD) caused by *Onchocerca volvulus*, a filarial nematode transmitted by blackflies of the genus *Simulium* [1]. In 2017, it was estimated that at least 220 million people required preventive chemotherapy against onchocerciasis, 14.6 million of the infected people had skin disease and 1.2 million had vision loss [2]. According to the Global Burden of Disease 2019 study, an estimated 19.1 million people are infected, with the disease being responsible for 1.23 [95% Uncertainty Interval = 0.77–1.82] million disability-adjusted life years (DALYs) [3]. More than 99% of cases occur in sub-Saharan Africa [2]. In Ghana, the at-risk population is greater than 2 million people [4], with onchocerciasis being endemic in 15 of its 16 regions [5].

The global health community, led by the World Health Organization (WHO) through a recently published NTD Roadmap for 2021–2030, aims at elimination (interruption) of transmission (EOT) for onchocerciasis, with 12 countries (about a third of all endemic countries) proposed to be verified for EOT by 2030 [6]. The inspiration for this target is drawn from Sustainable Development Goal 3 (SDG 3), which aims at achieving *Good Health and Well-Being for All*, the principle of leaving no one behind [7], the London Declaration on NTDs [8], and the recent Kigali Declaration on NTDs [9, 10].

The achievement of onchocerciasis EOT is strongly dependent on the success of national programmes delivering annual/biannual mass drug administration (MDA) of ivermectin in endemic communities [8]. Ivermectin is a safe and efficacious microfilaricide (i.e. clears the microfilarial progeny of the parasite), exerting also a temporary embryostatic effect (i.e. transiently reducing production of live microfilariae (mf) by the female adult worm) [11]. Since microfilarial production is resumed within 4–6 months following treatment and skin repopulation by mf can be substantial at 12 months post-treatment [11], annual MDA may not be sufficient to curtail transmission during the inter-treatment period, particularly in areas with high vector biting rates (e.g. hyperendemic areas), indicating the need for biannual treatment [8]. In addition to its microfilaricidal and embryostatic effects, repeated exposure to ivermectin may lead to a more permanent sterilizing effect [12] and/or to a macrofilaricidal effect (against the adult filariae) [13]. However, due to the long lifespan of the latter (10 years on average), there is a need for uninterrupted high geographical and therapeutic coverage of ivermectin MDA for at least 15–20 years (and possibly longer) in order to interrupt *O. volvulus* transmission in endemic areas [14]. Successes (with annual or biannual MDA) recorded in some foci of Mali, Senegal, Nigeria, Sudan and Uganda [15–18] paved the way to shifting from elimination of onchocerciasis as a public health problem (control) to EOT.

The success of ivermectin MDA programmes to achieve EOT strongly depends on sustaining high levels of treatment adherence, with systematic non-adherence (the proportion of the population never taking treatment) being one of the most important factors hindering progress [19]. However, in MDA programmes, the frequently reported metric is the ‘therapeutic coverage’, which refers to the proportion of total (or of eligible) population who received the drug, and not necessarily ‘adherence’, which refers to the proportion of eligible population who actually ingests the drugs consistently over multiple treatment rounds [20–22]. Studies in endemic communities have demonstrated that despite high reported MDA coverage, treatment adherence is far from ideal [20, 23]. (Although the term ‘compliance’ has been used in many studies, we adopt the term ‘adherence’ to better reflect an active process of participation by individuals in treatment programmes.^1^) Substantial proportions of ‘non-adherers’ can act as persistent infection reservoirs in endemic communities, hampering the achievement of EOT [19, 22–24].

In Ghana, persistence of *O. volvulus* infection and its associated clinical manifestations has been documented in the Bono Region despite 27 years of ivermectin treatment [5, 25], with biannual delivery of community-directed treatment with ivermectin (CDTI) since 2009 [26]. In fact, Ghana is a country formerly included in the Onchocerciasis Control Programme in West Africa (OCP) [26, 27]. During the OCP (1974–2002), vector control, through aerial larviciding, was implemented in the northern and central parts of the country [27]. When ivermectin was licensed for treatment of onchocerciasis in humans in 1987, Ghana was one of the first countries to implement MDA in 1995, via mobile teams [5]. Following the closure of the OCP, regions classified as Special Intervention Zones (SIZ; areas where microfilarial prevalence had remained above 50%), received further interventions after 2002. In Ghana, SIZs included an area in the Pru River basin, where CDTI continued yearly till 2012 [28].

Following decades of ivermectin MDA, an onchocerciasis impact assessment was performed in 2017 by the Ghana Health Service (GHS). Community surveys conducted during the assessment revealed that infection prevalence was unexpectedly high. In the Ottukrom and Kwanware communities in the Wenchi municipality of the Bono Region, where the microfilarial prevalence in 1995 was 54.2%, surveys revealed that the prevalence in 2017 was 29% [95% Confidence Interval, CI = 16.1–46.6%] (9/31 adults aged ≥ 20 years, examined by skin-snip microscopy) and the Ov16 seroprevalence among < 10-year-old children was 38% [95% CI = 20.8–59.1%] (8/21 children examined by Ov16 rapid diagnostic test, RDT). In Abekwae, in the Tain District, the microfilarial prevalence in 2017 was 13.9% (9/65) [95% CI = 7.5–24.3%] and the Ov16 RDT seroprevalence was 9.3% (7/75) [95% CI = 4.6–18.0%] [5, 25]. Low treatment adherence could be a factor contributing to the persistence of infection in these and nearby communities. With the interruption of MDA in 2020 due to the COVID-19 pandemic and subsequent resumption of NTD activities in 2021, there is a need for studies to inform the implementation of remedial action, such as educational and behavioural change campaigns by the GHS, other policy-makers and implementation partners, as well as to identify mitigating strategies to help programmes get back on track to achieve the EOT target. Therefore, this study aimed to investigate the extent of and factors responsible for non-adherence to ivermectin treatment in 13 endemic communities with persistent *O. volvulus* infection in the Bono Region of Ghana despite 27 years of ivermectin MDA.

## Materials and methods

### Description of the study area and population

**Fig. 1:**
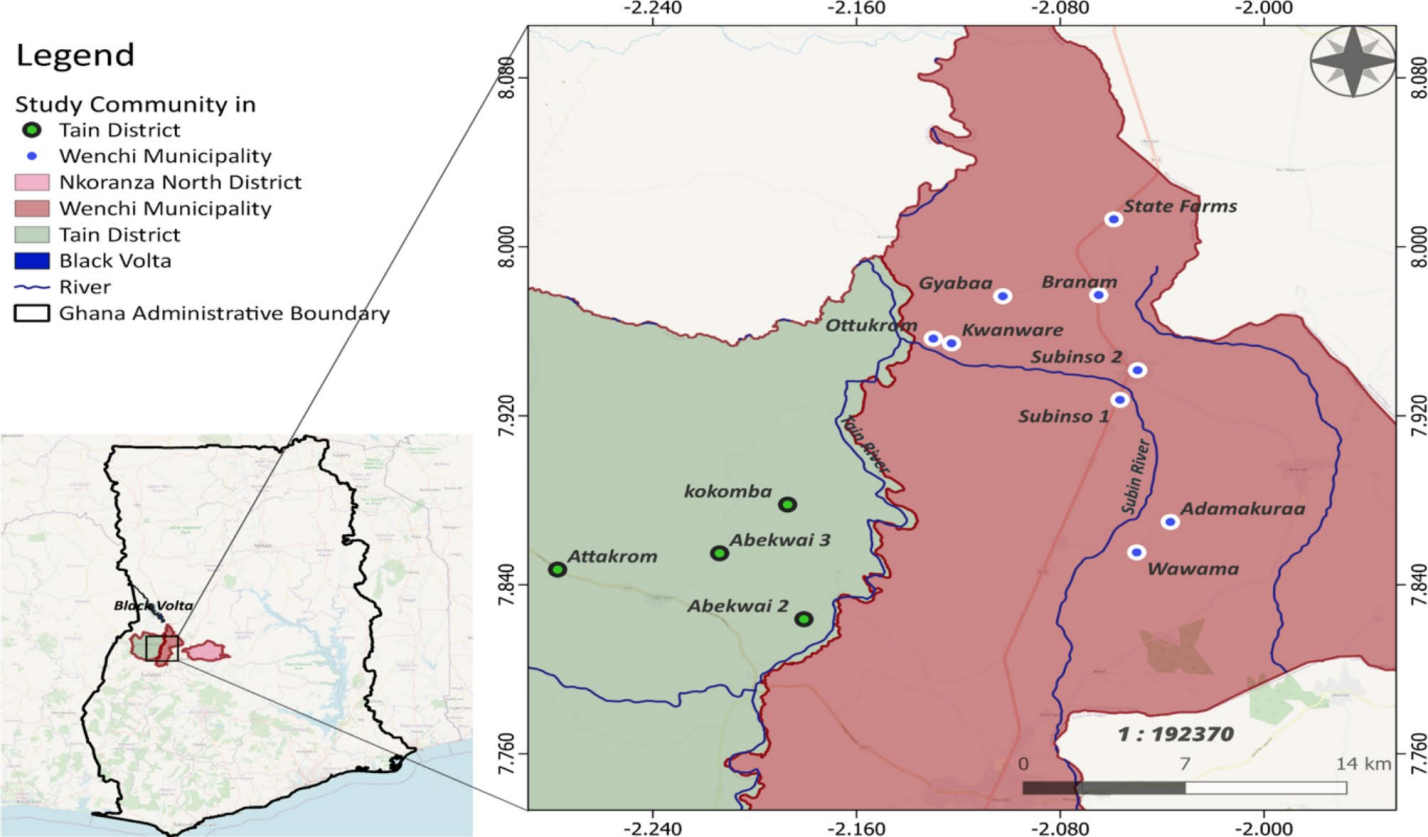
Map of study communities in the Tain District and Wenchi Municipality, Bono Region, Ghana

The study was conducted in 13 onchocerciasis-endemic communities in the Tain District and Wenchi Municipality of the Bono Region of Ghana. The Tain District has a total area of 1,829.85 square kilometres and a population size of 88,104 (50.6% females, 49.4% males) [29]. The Wenchi Municipality has a total area of 1,296.60 square kilometres and a population of 89,739 (50.9% females and 49.1% males) [30]. The study was performed in Abekwai 2, Abekwai 3, Attakrom and Kokomba in the Tain District, and in Adamukuraa, Branam, Gyabaa, Kwanware, Ottukrom, State Farms, Subinso 1, Subinso 2 and Wawama in the Wenchi Municipality (Fig. 1). The study map indicates that the study communities form two clusters. The first cluster consists of four villages: Abekwai 2, Abekwai 3, Attakrom and Kokomba. This cluster lies west of the main Tain River. The second cluster consists of nine villages: Adamukuraa, Branam, Gyabaa, Kwanware, Ottukrom, State Farms, Subinso 1, Subinso 2 and Wawama. This cluster is situated along the Subin River in the Wenchi Municipality. The two clusters have different vector control histories [5, 25]. The part of the Tain river from the village of Tainso to the junction with the Black Volta started vector control in 1976 as part of Phase II of the OCP, whereas the Subin river was part of the South-Eastern Extension of the OCP that became operational in 1988. The Subin river was under vector control from 1992 onwards though vector control may have started a few years earlier. In 1996, vector control ended in both the Tain and Subin rivers [5, 25].

The baseline *O. volvulus* (crude) microfilarial prevalence for Kwanware (in the Wenchi cluster) in 1989 was 48.1% [95% CI = 41.5–54.8%] and the community microfilarial load (CMFL) was 7.26 microfilariae per skin snip (mf/ss), indicating mesoendemicity according to OCP data [5]. In a survey conducted by the OCP in the year 2000, these values had decreased to 15.6% [95% CI = 10.0–23.6%] and 0.33 mf/ss, respectively, and in another survey conducted in 2012 (supported by the African Programme for Onchocerciasis Control (APOC) Trust Fund), the mf prevalence was 5.6% [95% CI = 2.2–13.6%] [5, 25]. All the other study communities in the Wenchi cluster lie within a 12 km radius of Kwanware and are located within the Subin River basin. For the Tain cluster, there are no data on pre-control microfilarial prevalence for our specific study villages. However, there is one nearby OCP village, Tainso, for which pre-control (crude) microfilarial prevalence is available for 1980 (40.7% [95% CI = 35.4–46.2%]) and which is situated within 4–10 km from each study village in the Tain cluster, also indicating mesoendemicity [5, 25].

The villages in both clusters have received MDA by mobile teams since 1994–1995, and CDTI since 1998 [5, 25]. Therefore, they have been under ivermectin MDA for nearly three decades. Since 2009–2010, the treatment strategy switched from annual to biannual ivermectin MDA in Tain and subsequently in Wenchi [5, 25]. In 2020 however, the two rounds of MDA were missed due to the COVID-19 pandemic, in keeping with the WHO’s directive to halt all MDA campaigns [31].

### Ethics approval and consent to participate

Ethical approval for this study was obtained from the Committee for Human Research and Ethics of the University of Energy and Natural Resources in Sunyani, Ghana, West Africa (Approval number: CHRE/AP/012/021). The study procedures were explained to the participants in the local dialect (*Twi*). For children and adolescents below 18 years, informed written consent was given by their parents/legal guardians. The informed consent forms were in English and the content was explained in the local language to participants who could not read or write in English. For participants who were non-literate (cannot read and/or write), the study was explained to them in the presence of a chosen literate witness from the community. The witness then signed the consent form whilst the non-literate participant thumb-printed it to indicate their willingness to participate. In addition, assent was sought for participants aged 10–17 years to affirm their willingness to participate in the study. Participants were informed that they had the option of withdrawing at any stage of the investigations, without any consequence. All procedures were performed in accordance with the Declaration of Helsinki.

### Study design and recruitment of study participants

The study adopted a mixed-methods, cross-sectional design. Quantitative and qualitative methods were used to collect data in August 2021 to determine the extent and factors that account for non-adherence to ivermectin treatment in the study communities. The study was performed in the framework of a larger project to determine the impact of the interruption in MDA due to COVID-19 on EOT targets in Ghana. One or two weeks prior to conducting the surveys, announcements were made in the study communities via the community information centres or with the aid of a ‘gong-gong’ beater (a local means of giving information to the community residents where the ‘beater’ moves around sounding a metallic instrument ‘the gong-gong’ whilst intermittently shouting out the information). Community residents were informed to gather at chosen social centres in the community. Once they were gathered, the study was explained in English and then in the local ‘*Twi’* language in the presence of the village elders and community members at the designated rendezvous point in the community. All individuals aged ≥ 10 years were invited to participate in the study and were recruited once they agreed and consented as described above.

### Quantitative methods

The questionnaire (Additional file 1, Instrument 1) that was used in this study was deployed on the KoboCollect App linked to an online KoboCollect server account (www.kobotoolbox.org) on electronic tablets. The questionnaires were pre-tested in communities neighbouring the study areas and administered by trained members of the research team. The questionnaire consisted of 38 questions including socio-demographic characteristics such as age, gender, level of education, religion, ethnicity, marital status and duration of residence in the community. The questionnaire also included questions to assess adherence to ivermectin treatment and perceptual factors influencing such adherence. To ensure that participants understood the questions, local language and local terms for technical words were used. For instance, ivermectin was referred to as ‘*nko aduro’* (meaning ‘oncho’ drug); blackflies as ‘*nnodowa*’ and onchocerciasis as ‘*nkoyare*’ (meaning ‘oncho’ disease).

### Qualitative methods

The study held focus group discussions (FGDs) among community residents and in-depth interviews using semi-structured guides with open-ended questions with community drug distributors (CDDs) and community leaders (Additional file 1, Instrument 2) to further explore the factors responsible for non-adherence. CDDs play a crucial role in the implementation of CDTI. Consisting of volunteers selected by community members to distribute ivermectin [32], they are trained and re-trained every 1–2 years to deliver treatment in the community and educate community members on health issues [33]. For the qualitative study, a total of 10 CDDs (Additional file 1, Table S1) and 13 community leaders (one per community) were interviewed. A total of 48 FGDs involving study participants from the quantitative study were also performed.

FGDs were held with 4–6 persons per group in each community. All the interviews were audio-recorded and transcribed verbatim and subsequently analyzed. In all, a total of 10 CDDs and 13 community leaders were interviewed and 48 FGDs were held. The community leaders were either the chief of the communities or their representatives. To reduce social desirability bias, FGDs did not include CDDs or opinion leaders as they could influence the responses of participants. The CDDs and opinion leaders were interviewed during separate in-depth (key informant) interviews. To reduce shyness bias, warm-up questions were used to start the FGD discussions.

### Sample size calculation and statistical analysis

The sample size of the study was computed using the Slovin’s formula [34], a non-parametric approximation used to calculate the sample size necessary to achieve a certain confidence level when sampling a population for which there is not enough information about the population’s behaviour or the distribution of a behaviour of interest,

$$n=\frac{N}{\left(1+N{e}^{2}\right)}$$

where $n$ is the sample size, $N$ is the population size, and $e$ is the margin of error. The population size was estimated at 15,000 and the margin of error was set at 5%, yielding a minimum sample size of 390. However, we included a total of 510 individuals, and obtained complete information for 483. We recruited an average of 40 individuals per community, the typical sample size per village for most epidemiological studies on onchocerciasis as reported by the WHO [35].

Data analyses were done using Graph Pad Prism statistical software© (Version 5) and Jamovi (version 2.3.19.0). Descriptive statistics were used to present simple frequencies of the socio-demographic variables (gender, age, education, marital status, religion, ethnicity, duration of stay in the community), adherence to ivermectin treatment and reasons for non-adherence. Chi-squared tests were used to investigate associations between various categorical variables. An onchocerciasis perception score was created to quantify people’s knowledge and views of onchocerciasis. The responses to the first three questions concerning knowledge about onchocerciasis (“What do you know about onchocerciasis?”) were coded as 1 if correct and 0 if wrong (Additional file 1, Instrument 1). We summed up the scores for these three questions to obtain the onchocerciasis perception score ranging from 0 to 3. For multivariable analysis, we constructed two logistic regression models (one for ivermectin intake in 2021, and the other for systematic intake of ivermectin during MDA) with generalized estimating equations (GEE) to account for the correlation of responses from participants in the same community. This was done using the ‘geeglm’ function in the R-package ‘Geepack’ and the village of residence was introduced as the clustering variable. Purposefully selected covariates for the final multivariable analyses included age, gender, education level, duration of stay in the village, and onchocerciasis perception score. The Quasi-information criterion (QSI) was used for model selection.^2^

All interviews and FGDs were audio-recorded and transcribed verbatim. A three-stage thematic coding approach was undertaken, using the interview topic guide to help structure the analysis. This was complemented by a more iterative approach which drew on aspects of grounded theory and allowed for new themes and ideas to develop from the interviews and FGDs [21]. Methodological triangulation (use of data from both quantitative and qualitative techniques) was used to corroborate the findings obtained by both methods. We also utilized data triangulation (use of data from community residents, CDDs, and community leaders) to corroborate the findings of the study. Lastly, we employed investigator triangulation where different authors independently analysed the results to confirm findings and reduce the level of bias.

## Results

### Quantitative study

#### Socio-demographic characteristics of study participants

The quantitative study recruited a total of 510 participants consisting of 274 (53.7%) females and 236 (46.3%) males with a median age of 32 years [Interquartile Range, IQR, 30 (20–50)] (Table 1). A large proportion (43.9%) had not received any formal education and 48.2% were born and resided in the village (Table 1).

**Table 1:** Socio-demographic information of 510 study participants (unless otherwise stated)

Variables (no. of respondents)	Overall (n = 510)
**Gender (510)**	
Female	274 (53.7%)
Male	236 (46.3%)
**Age (years) (483)**	
Median (IQR)	32.0 (20–50)
Range	10–92
**Current marital status (483)**	
Divorced	12 (2.5%)
Married	297 (61.3%)
Single	99 (20.5%)
Widow/Widower	25 (5.2%)
Not applicable (Children aged 10–15 years)	51 (10.6%)
**Education (510)**	
No formal education	224 (43.9%)
Basic School	136 (26.7%)
Junior High/Secondary School	113 (22.2%)
Senior High/Secondary School	29 (5.7%)
Tertiary	8 (1.6%)
**Duration of residence/stay in village (510)**	
Born and resided in the village	246 (48.2%)
Not born in the village but resided in the village for < 1 year	29 (5.7%)
Not born in the village but resided in the village for 1–3 years	39 (7.6%)
Not born in the village but resided in the village for 4–7 years	38 (7.5%)
Not born in the village but resided in the village for 8–11 years	62 (12.2%)
Not born in the village but resided in the village for ≥ 12 years	96 (18.8%)

#### Ivermectin adherence

Of the 510 participants, 320 (62.8%) declared that they had adhered to each treatment round. For the 483 participants with complete information, this value was 63.6% and 176 (36.4%) reported that they had missed at least one round of ivermectin despite being eligible at the time of distribution (Table 2). Up to 5% stated they had never taken ivermectin in any MDA round (systematic non-adherers), and 28.8% reported that they did not take ivermectin during the last round of MDA (in March 2021).

**Table 2:** Ivermectin intake among the study participants with complete information

Variables (no. of respondents)	Overall (n = 483)
**Took ivermectin at least once in an MDA round (n = 483)**	
No	24 (5.0%)
Yes	459 (95.0%)
**Took ivermectin consistently in each round since becoming eligible (n = 483)**	
No	176 (36.4%)
Yes	307 (63.6%)
**If Yes, how many times since the first ivermectin intake? (n = 307)**	
Once	12 (3.9%)
Twice	11 (3.6%)
Thrice	21 (6.8%)
Four times	9 (2.9%)
Five times	6 (2.0%)
>5 times	245 (79.8%)
Cannot remember	3 (1.0%)
**Took ivermectin during the last round, March 2021 (n = 483)**	
No	139 (28.8%)
Yes	344 (71.2%)

#### Reason for not taking ivermectin

Participants gave various reasons for non-adherence to ivermectin. Of the 24 (5%) who had never taken ivermectin before, 41.7% cited the fear of side-effects as a reason for non-adherence, whilst 37.5% were absent during the MDA campaigns (Table 3). For those who did not take ivermectin in the last round of MDA (139), 46% were absent, with the remainder missing the drug intake due to drug distribution challenges, pregnancy, having previously experienced side-effects, refusal for various perceptual reasons and others.

**Table 3:** Reasons for not taking ivermectin

Variables (no. of respondents)		n (%)
**Why have you never taken ivermectin (n = 24)**		
Fear of side-effects		10 (41.7%)
Absent		9 (37.5%)
Drug not available in community		3 (12.5%)
Onchocerciasis is not an important disease		2 (8.3%)
**Why did you not take ivermectin in the last round? (n = 139)**		
Absent		64 (46.0%)
Distribution challenge		33 (23.7%)
Pregnancy		16 (11.5%)
Previous side-effects		11 (7.9%)
Refused		9 (6.5%)
Other reason		6 (4.3%)
**If absent above, why? (n = 64)**		
Travelled		54 (84.4%)
Was at work		6 (9.4%)
Was in school		4 (6.3%)
**If distribution challenge, specify (n = 33)**		
Treatment available but not reached by CDD		16 (51.6%)
Treatment not available		17 (48.4%)
**If refused above, why? (n = 8)**		
Fear of side-effects		6 (75.0%)
Prefer traditional medicine		1 (12.5%)
The purpose of drug was not explained to me		1 (12.5%)
**If other reason, specify (n = 6)**		
Drug got misplaced		2 (33.5%)
Forgot to swallow drug when collected		2 (33.3%)
Onchocerciasis is not an important disease		1 (16.7%)
Other sickness		1 (16.7%)

#### Perceptual factors influencing ivermectin intake

The study also investigated some perceptual reasons for non-adherence to ivermectin and the findings are summarized in Additional file 1, Table S2. About 9.2% (47/510) of participants did not believe that onchocerciasis is a serious disease whilst 7.1% (33/462) believed that the best treatment for onchocerciasis is traditional medicine. Also, 12% (61/510) of study participants believed that ivermectin intake is problematic. This notwithstanding, based on the participants for whom information was available, 80% acknowledged that they took the drug willingly for their own health while another 14.4% took the drug because someone encouraged them to do so. The median onchocerciasis perception score was 1.0 (IQR: 1.0–2.0) on a scale of 0 to 3.

#### Independent predictors of ivermectin intake

The multivariable GEE logistic regression models found that gender, education level, and perception of onchocerciasis were associated with ivermectin intake. Regarding adherence to ivermectin MDA in 2021 (Table 4), being male and having a high onchocerciasis perception score significantly increased the odds of ivermectin intake. Having a higher education level (secondary education and above) significantly decreased the odds of having taken ivermectin in the March 2021 MDA round. Neither age nor duration of residency in the community were statistically significant.

**Table 4:** Results of the final multivariable regression model investigating predictors of ivermectin intake during the 2021 MDA round

Covariates	Odds ratio (95% CI)	*P*-value
Age in years	0.999 (0.984–1.02)	0.928
Male gender	1.780 (1.21–2.60)	0.003
Education level		
None Primary Secondary and above	Reference 0.796 (0.410–1.54) 0.550 (0.320–0.945	Reference 0.499 0.030
Duration of stay in the village in years	1.010 (0.992–1.02)	0.456
Onchocerciasis perception score	1.430 (1.19–1.72)	< 0.001
*n = 468 after removal of missing values* *Quasi Information Criterion (QIC) = 540.46*		

Similarly, the odds for systematic (consistent) intake of ivermectin during MDA rounds significantly increased with high onchocerciasis perception scores. Having a secondary education level and above significantly decreased the odds of systematically adhering to ivermectin intake (Table 5).

**Table 5:** Results of the final multivariable regression model investigating predictors of systematic ivermectin intake during MDA

Covariates	Odds ratio (95% CI)	*P*-value
Age in years	1.000 (0.988–1.02)	0.743
Male gender	1.440 (0.947–2.19)	0.088
Education level		
None Primary Secondary and above	Reference 1.040 (0.517–2.10) 0.606 (0.423–0.867)	Reference 0.907 0.006
Duration of stay in the village in years	1.010 (0.997–1.03)	0.122
Onchocerciasis perception score	1.230 (1.03–1.48)	0.023

*n = 468 after removal of missing values*

*Quasi Information Criterion (QIC) = 603.22*

### Qualitative study

#### Reasons for not taking ivermectin

The findings from the quantitative study were confirmed in the qualitative study.

#### Side-effects/fear of side-effects

Some participants explained that they stopped taking ivermectin due to having experienced side-effects or because of the fear of side-effects from stories of side-effects experienced by others. This was corroborated during interviews with the CDDs:

*‘I stopped taking the drug because I think it does not agree with my body. Each time I swallowed the drug, I had boils and rashes on my skin, so I stopped.’ (FGD male participant, Branam).*

*‘Yes, there are some people who don’t take the drugs, or sometimes just take it from you and will not swallow it because they fear that it will make them to become swollen or they will not be able to perform in bed or can’t go the farm because of the oncho drug’s effect.’ (CDD, interview, Abekwai 3).*

*‘For this community a lot of people have the notion that if you take the oncho drug, you will have reactions, so for them, even if you distribute the drug three times in the year, they will never take it.’ (CDD, interview, Adamukuraa).*

### Absence due to travelling

Some people only missed ivermectin intake when they travelled. This was especially a problem in the rural villages because students had to travel from their villages to their schools which were usually boarding schools far away from the villages. Adults, especially males, also frequently travel to the larger towns for work-related activities and sometimes miss the intake of ivermectin:

*‘I have never swallowed the drug because I keep moving between different villages. By the time it comes to one village then I will be in the next village.’ (FGD male participant, Subinso 2).*

*‘The only time I do not take the drug is when I travel out of the village, otherwise, I always take it.’ (FGD Female Participant, Branam).*

#### Distribution challenges

Other reasons for not taking ivermectin were distribution challenges, misplacement of drugs, and being sick at the time of MDA:

*‘The last time, I travelled and my wife collected my drugs for me. When I came back, she searched for the drugs and could not find them. It was missing so I did not get the drugs to swallow them.’ (FGD Male participant, Branam).*

*‘The problem is with those who are not around. You give the drug to the family member to give to them and you don’t know if they actually swallow the drug.’ (Community Leader, interview, Abekwai 3).*

*‘The problem is that because I travel a lot, someone will usually collect the drug for me but before I come back, they cannot find it.’ (FGD male participant, Kokomba).*

#### Ineligibility due to pregnancy

Concerning pregnancy as a reason for not taking ivermectin, the qualitative study corroborated the findings of the quantitative study. A FGD participant remarked:

*‘Unfortunately, during those periods, whenever they brought the drug, I would be pregnant and was told I cannot take it due to pregnancy. He (CDD) did not give it to me to put down till after pregnancy.’ (FGD female participant, Abekwai 2).*

This was corroborated by CDDs:

*‘The only people that I usually do not give ivermectin to in this village include pregnant women…they are always excluded from the distribution. When it happens like that, they are not usually happy, but I have to work according to instructions.’ (CDD, in-depth interview, Kwanware).*

#### Desire to drink alcohol

Some participants confirmed that sometimes their desire to drink alcohol was also a reason for not taking ivermectin. This was especially common among young men:

*‘A major problem with ivermectin intake in this community is that a lot of young men like alcohol too much. So, when you give them the drug and tell them not to take alcohol, they collect the drug and go and put it somewhere without swallowing it. Maybe you (health authorities) should consider a new drug that does not require that you abstain from alcohol.’ (Community leader, interview, Attakrom).*

*‘There was one case here. You know they (health authorities) usually tell us that when you take alcohol, you cannot take the drug. This person did not listen and took the alcohol after taking the drug. He nearly died. So, after that incident whenever they bring it (ivermectin), he will not take it.’ (FGD male, Abekwai 2).*

#### Role of CDDs and community leaders in motivating people

The CDDs interviewed were all males with an average of 20 years of experience in CDTI. The community leaders were also all males. Other duties of CDDs aside of ivermectin distribution included assisting in distribution of vaccines by the health authorities. For all the 13 communities, the main mode of distribution of ivermectin was door-to-door and using the directly-observed treatment method. The impact of CDDs on adherence was observed:

*‘In my village here, there was this young man, a friend of mine who was regularly collecting the drug, but was never taking it for fear of side effects. When I found out, I spoked to him and since then he has been swallowing the drug whenever I bring.’ (CDD, interview, Abekwai 2).*

*‘For me, I have attended many workshops and watched a lot of videos on oncho disease. Because of this, I am usually passionate about the way I approach the work. I have done this work for over 20 years, travelling about 14 miles to some communities and usually go door-to-door to distribute the drug.’ (CDD, interview, Gyabaa).*

#### Perceptual factors influencing ivermectin intake

The statements of the participants during the qualitative study corroborated the findings from the quantitative study:

*‘I know of a number of my friends who do not take drugs. One of them especially says that he is not convinced the disease is even there because he has not seen someone with it (the disease) before. He does not also believe in the education that they (health authorities) make about the disease so he will not take the drug.’ (FGD male participant, Branam).*

*‘Some people think that they are not sick so there is no reason to swallow the drug, so when they are given the drug by the CDD, they collect it and throw it away.’ (FGD Female, Abekwai 3).*

*‘Me, I do not know whether or not the drug is effective because I don’t see changes in my body.’ (FGD Male, Abekwai 2).*

#### Community perspectives on how to alleviate the fear of treatment and improve adherence

Factors identified to improve adherence to ivermectin treatment included providing more resources to CDDs, improving trust in the health authorities, education on benefits of swallowing the drug, using the directly-observed treatment method and increase education on the risk of the disease.

*‘As the Drug distributor for 4 different communities, always, I have to travel a distance of about 14 miles to and from communities. This is difficult to do on the bicycle they gave us which is even broken and the money paid is too small for lorry fare. This usually affects our distribution work and it will help if they (Health authorities) can do something about it.’ (CDD, interview, Branam).*

*‘I know a number of friends who do not take drugs. One of them says that he is not convinced the disease is even there because he has not seen some before.’ (FGD male participant, Branam).*

‘*Here, you are given a cup of water and you are observed to swallow the drug so everyone takes the drug.’ (FGD male, Abekwai 2).*

*‘They say that we should swallow the drug, it will help us, and that is why I also swallow, though I don’t see anything.’ (FGD female, Adamukuraa).*

## Discussion

This study investigated adherence to ivermectin treatment in 13 endemic communities with persistent onchocerciasis in the Bono Region of Ghana which have been under MDA for nearly three decades.

The results showed that 36.4% of the study participants had missed at least one round of ivermectin despite being eligible at the time of distribution; 28.8% did not take ivermectin in the last (March 2021) round, and 5% had never taken the drug (systematic non-adherers). The success of onchocerciasis elimination is highly dependent on the adherence to treatment in endemic populations during MDA rounds [8, 14, 19]. This is especially important because non-adherers, and particularly systematic non-adherers (never treated) may act as infection reservoirs in the communities, contribute to transmission and derail efforts to achieve EOT [22]. Although the problem of non-adherence is not unusual, the observed level of non-adherence with the last round of MDA at the time of the study (29%) is relatively high. For instance, 19% of 308 study respondents reported not taking ivermectin during the last (9th ) round of annual MDA in the Kabo area (Gambella Region) of southwestern Ethiopia in 2012 [36], and a study in Uganda reported that 21% of 839 people interviewed had not taken ivermectin during the last (10th ) round of annual MDA in 2002 in the Bushenyi District [37]. However, the interviews in Ethiopia were conducted three weeks after MDA and those in Uganda took place two months after treatment, whilst our study was done five months after the first of the two rounds of biannual treatment in 2021, following the disruption to all treatment campaigns and NTD activities caused by the COVID-19 pandemic.

The proportion of respondents in our study who reported consistently taking ivermectin during each round since becoming eligible was 63.6%, with 79.8% taking treatment for at least five rounds. In the Bench Maji Zone of southwestern Ethiopia, the proportion of 553 respondents (aged ≥ 15 years) consistently adhering to treatment over five years of biannual MDA (10 rounds) was 65.3% [24]. In the Centre, West and Littoral regions of Cameroon 57.8% of those interviewed (aged ≥ 10 years) declared having taken treatment each time during the last 5 MDA rounds (2010–2014), with 9.8% of systematic non-adherers (never treated) [38].

Among the systematic non-adherers in our study, the most common reason given for never taking treatment was the fear of side-effects reported by others in the community, closely followed by being absent at the time of drug distribution. For those who did not take ivermectin during the March 2021 round, the main reason was not being in the community at the time of MDA (Table 3). Common side-effects declared during FGDs and interviews were oedema (of limbs, face, penis), boils, rashes and lesions, loss of libido, general malaise, musculoskeletal pains, immobility, dizziness and headaches. Most participants, however, admitted that these side-effects often resolved within 2–5 days. Fear of side-effects has also been reported as a major reason for not adhering to treatment in previous studies conducted in Ghana [23, 39]. In the Upper Denkyira East Municipality, Central Region, fear of side-effects was reported by a decreasing fraction of respondents (76%, 23%, 16%) in 2002, 2006 and 2013, respectively, but the proportion taking treatment did not follow a correspondingly increasing trend (76%, 64%, 79%), yielding a mean rate of non-adherence to ivermectin intake of 27% for those years [39], in line with our 29% for 2021.

Experiencing severe adverse effects (SAEs) in past treatment rounds, or fear of SAEs experienced by others negatively impacts on community participation in and treatment adherence to ivermectin MDA, particularly in onchocerciasis-loiasis co-endemic areas [19, 21, 40, 41]. A study in South-West Cameroon demonstrated that the fear or past experience of side-effects associated with ivermectin treatment was the main reason for non-adherence despite the fact that the area was at relatively low risk of loiasis and that no fatal encephalopathies had been reported [19]. Wanji et al. [40], also working in onchocerciasis-loiasis co-endemic areas of South-West Cameroon, documented that the proportion of systematic non-adherers was nearly 16%, and that although the majority (40%) of the study participants (2,364 people) had taken the drug 1–3 times, only 18% had taken it at least 7 times (quantified by the participants’ oral declaration). There was also a clear correlation between treatment adherence and levels of microfilarial infection, with the highest prevalence (60%) found among the systematic non-adherers and the lowest (34%) among those who had taken ivermectin ≥ 7 times [40]. A relationship between treatment adherence and microfilarial prevalence was also reported in the Bench Maji Zone of southwestern Ethiopia (without *Loa loa*), where the prevalence among those who had missed at least one MDA round for the past 15 years of CDTI was 10% compared to 3% in those who had consistently taken ivermectin. The proportion of participants refusing treatment (systematic non-adherers) was 5.6% (31/553) [42].

Absenteeism due to travelling was also one of the most frequently recorded reason for not taking ivermectin in our study area (both among systematic non-adherers and among those who had missed the March 2021 treatment round, Table 3). In fact, most community residents had not been not born in the villages (54.2%, Table 1) but had made their settlements in the communities for various reasons, such as farming. Such persons usually return to their ‘hometowns’ during festive periods and also after major farming seasons. In a study by Hamilton et al., in the then Brong Ahafo region of Ghana, absence during drug distribution was the major contributor (52%) to missed treatments [43]. Being absent at the time of MDA was also reported in Cameroon and two studies in Ghana, where it was given as a reason for not taking ivermectin in, respectively, 37%, 32% and 30% of those who had missed the last treatment round being evaluated [21, 23, 27]. The study by Senyonjo et al. [21] in Cameroon remarked that the lowest levels of adherence, recorded for young adults (aged 20–34 years), could be due to increased work and mobility amongst this age-group, while lower adherence levels among those who had moved into the village in the last five years compared to longer-term residents, could be owing to lack of awareness of the MDA campaign and/or the risks of onchocerciasis.

In our study, age was not significantly associated with treatment adherence (Tables 4 and 5), in contrast with other studies which found age to be positively [38] or negatively [36] associated with taking ivermectin. Our study found that men were more likely to have taken ivermectin compared to women (Table 4), in agreement with Agyemang et al. [39], also in Ghana, whilst no statistically significant differences in adherence between males and females were reported in other studies [19, 20, 23, 24, 38, 43]. Our lower adherence among women contrasts with the notion that women tend to have better health-seeking behaviours than men [44]. However, for young women of reproductive age (WRA), a major reason not to take ivermectin was pregnancy. Some women expressed concern that because they frequently undergo the cycle of pregnancy, motherhood, breast-feeding and back to pregnancy, they missed several ivermectin treatment rounds. In the study by Forrer et al., refusal of ivermectin by WRA based on the belief that it leads to miscarriages was highlighted as a factor contributing to non-adherence in this population group [19]. To date, pregnant women are excluded from MDA with ivermectin programmes for onchocerciasis and other helminthiases (such as lymphatic filariasis). Ivermectin has been assigned to pregnancy risk category C (risk cannot be ruled out) [45] by the USA Food and Drug Administration (FDA), and therefore the manufacturers consider ivermectin contraindicated in pregnancy. However, in their systematic review of the safety of oral ivermectin during pregnancy, Nicolas et al. found that no study reported neonatal deaths, maternal morbidity, preterm births or low birthweight [46]. Exclusion of pregnant women may help sustain a substantial infection reservoir and deprive a vulnerable population of potential benefits, as there are indications that treating *O. volvulus-*infected women may improve pregnancy outcomes and reduce the risk that their children develop onchocerciasis-associated morbidities [47]. Therefore, further studies are needed to investigate the safety and potential benefits of ivermectin for this vulnerable population. It should also be investigated whether, when taking into account pregnancy, being female remains significantly associated with lower ivermectin adherence.

Another reason for non-adherence identified in our study was the desire to drink alcohol, especially among men. Alcohol consumption in some of the study communities was relatively high as it is usually considered *‘men’s water’* in some villages. A typical routine for some young men in several communities is to ‘*cut a little alcohol’* for appetite, libido and energy to work. In the Democratic Republic of Congo (with co-endemic loiasis), alcohol intake 24 h prior to ivermectin treatment was significantly associated with neurological SAEs [41]. However, in a study by Homeida et al., a locally brewed alcoholic beverage given with ivermectin did not cause changes in the plasma pharmacokinetic parameters of ivermectin, suggesting that alcohol intake is unlikely to be a contributory factor in the development of the SAEs that can occur following ivermectin treatment of individuals co-infected with loiasis [48].

Our multivariable analysis indicated that individuals with better perception and knowledge of the disease and the beneficial effects of MDA had better health-seeking behaviours and were more adherent to ivermectin intake. These results were echoed in both the quantitative and qualitative findings of our study, and are in agreement with the results of other studies [23, 38]. We also found that people with lower levels of formal education tended to be more adherent to MDA than those who had attained higher levels (Tables 4 and 5). This finding is consistent with a study conducted in Cameroon and Nigeria, which discussed that those with higher education may be more mobile and harder to reach during MDA campaigns [49]. For instance, people who are undergoing formal education in secondary and tertiary schools (mostly located in urban areas) may have to travel outside their communities for several months of the year for school, increasing their chances of missing MDA rounds.

The low CDD : population ratio (about 1 : 1,000) in our study communities is of concern (Table S2). The travel time between communities explains why certain individuals may not be reached. The reasons for the low numbers of CDDs in endemic communities in Ghana may include lack of cooperation and/or multiple demands by community members, scarce resources for the work and insufficient financial incentives, leading to the resignation of CDDs [32]. The study of Agyemang et al. [39] in Ghana pointed out that CDDs are required to complete the distribution of ivermectin in the entire village, covering all households, within just seven days irrespective of the size of the catchment area. As the success of MDA depends, to a large extent, on the essential role of CDDs [32], it is important to put in place remedial actions to address these challenges.

This study had a number of limitations. Study participants were not randomly selected. Therefore, results need to be interpreted with caution as the findings may not be representative of all the persons living in the study communities. Also, a social desirability bias may have influenced the responses to the questions, although participants were made aware that the responses had no punitive implications for them whatsoever. To reduce recall bias, we performed the study five months after the last round and immediately before the next round (which took place in August 2021 in an effort to mitigate the impact of the missed MDA treatment rounds due to COVID-19). However, other adherence studies were conducted much sooner after the last treatment round being evaluated [36, 37]. Notwithstanding, the particular features of ivermectin tablets (small-size, white-colour tablets), their availability only during MDA campaigns, and their distribution accompanied by sensitization and door-to-door delivery, provide a strong reference for people to recall [50]. Another limitation is that we did not interview healthcare workers at district, municipal, regional and national levels, as we focused on community-level factors that affect treatment adherence. Finally, we did not interview community-based organizations, which might have provided valuable information on treatment adherence in their communities.

## Conclusions and recommendations

In line with other studies where onchocerciasis persists despite prolonged CDTI, the proportion of eligible people who had missed at least one round of ivermectin, or who did not take ivermectin in the last treatment round being evaluated was relatively high. However, the proportion of systematic non-adherers (around 5%) is in broad agreement with that recorded in areas not co-endemic with loiasis. Otabil et al. had documented therapeutic coverage levels (of total population) of about 80% in the study communities, with the proportion of people absent or refusing treatment ranging between 2% and 5%, as recorded by the CDDs [5]. However, our study indicates that actual ivermectin intake (swallowing of the tablets) is lower for the reasons revealed and discussed above. Modelling studies have compared the impact of various levels of therapeutic coverage and systematic non-adherence on the duration of onchocerciasis treatment programmes, concluding that minimising the proportion of the population which never takes treatment is fundamental to reach elimination targets [8, 14]. Non-adherers constitute potential sources of infection to the blackfly vectors during their inter-treatment periods, which can be long and allow for substantial skin repopulation by *O. volvulus* mf if several rounds of treatment are consecutively missed by the same individuals. This, together with the fraction of the population who are never treated, jeopardize the achievement of EOT.

We propose the following recommendations:

1. Intensified and targeted community educational campaigns should be implemented to address fears of side-effects, also taking seriously the concerns of CDDs regarding the identification and management of side-effects in their training sessions, as suggested by Agyemang et al. [39]. There is also a need to implement an effective SAE surveillance system during MDA campaigns, similar to that of the Expanded Programme on Immunization [23, 51].
2. Consideration should be given to increasing the period allocated for MDA, and whenever possible to improving its timing with the aim to minimise the number of absentees during the treatment rounds (considering also that MDA timing should be optimised to coincide with transmission seasonality [14]). This could also assuage CDDs’ concerns about having to complete all treatment activities in only a week regardless of the size of the catchment area [39].
3. Provision of resources for CDDs should be improved and efforts to increase the trust of the populations in the health authorities should be made. Greater emphasis should be placed on explaining clearly the risks of onchocerciasis and the benefits of treatment, and wherever possible the tablets should be delivered using the directly-observed treatment method and via door-to-door distribution (as adopted during the resumption of MDA following COVID-19).

In conclusion, our results highlight a pressing need for the Ministry of Health of Ghana, the GHS, other stakeholders, policy-makers and implementation partners to develop targeted remedial strategies including educational campaigns and behavioural and perceptual change campaigns regarding onchocerciasis and its treatment if the country is to achieve EOT by 2030.

### Electronic supplementary material

Below is the link to the electronic supplementary material.

Supplementary Material 1

## Data Availability

The datasets used and/or analyzed during the current study are available via the ***Zenodo*** repository (https://zenodo.org/record/7654986#.Y_J0B7TP06A).

## Abbreviations

APOC: African Programme for Onchocerciasis Control
CDD: Community drug distributor
CDTI: Community directed treatment with ivermectin
CI: Confidence interval
CMFL: Community microfilarial load
COVID-19: Coronavirus disease 2019 (SARS‑CoV‑2)
DALY: Disability-adjusted life year
EOT: Elimination of transmission
FDA: Food and Drug Administration (of the USA)
FGD: Focus Group Discussion
GEE: Generalized estimating equation
GHS: Ghana Health Service
IQR: Interquartile range
MDA: Mass drug administration
mf: microfilariae
NTD: Neglected tropical disease
OCP: Onchocerciasis Control Programme in West Africa
Ov16: *Onchocerca volvulus* antigen 16
QIC: Quasi Information Criterion
RDT: Rapid diagnostic test
SAE: Severe adverse effect
SDG: Sustainable Development Goal
SIZ: Special Intervention Zone
WHO: World Health Organization

## Footnotes

1 Adherence is an active process in which a patient takes responsibility for their overall well-being, while compliance is a passive behaviour in which a patient is following a list of recommendations. Mir TH. Adherence versus compliance. HCA Healthc J Med. 2023;4: 219–220.

2 The quasi information criterion (QIC) has been proposed as an alternative to Akaike’s information criterion (AIC) for regression analysis based on GEE (Pan W. Akaike’s Information Criterion in Generalized Estimating Equations. Biometrics.2001;57:120–25).
