# Supplementary material for: Non-adherence to ivermectin in onchocerciasis-endemic communities with persistent infection in the Bono Region of Ghana: a mixed-methods study
Source: BMC Infect Dis. 2023 Nov 16;23:805. doi: 10.1186/s12879-023-08806-8 (PMC10655298; doi:10.1186/s12879-023-08806-8)
Supplement: Supplementary file 1 — Supplementary Material 1 [file 12879_2023_8806_MOESM1_ESM.docx]

**Additional file 1**

**Non-adherence to ivermectin in onchocerciasis-endemic communities with persistent infection in the Bono Region of Ghana: A mixed-methods study**

Kenneth Bentum Otabil^1,2,3*^, María-Gloria Basáñez^4^, Blessing Ankrah^1^, Emmanuel John Bart-Plange^1^, Theophilus Nti Babae^1^, Prince-Charles Kudzordzi^1^, Vera Achiaa Darko^1,5^, Abdul Sakibu Raji^2^, Lydia Datsa^6^, Andrews Agyapong Boakye^7^, Michael Tawiah Yeboah^8^, Joseph Nelson Siewe Fodjo^3^, Henk D. F. H. Schallig^9^, Robert Colebunders^3^

*^1^Consortium for Neglected Tropical Diseases and One Health, Department of Biological Science, School of Sciences, University of Energy and Natural Resources, Sunyani, Bono Region, Ghana*

*^2^Department of Biological Science, School of Sciences, University of Energy and Natural Resources, Sunyani, Bono Region, Ghana*

*^3^Global Health Institute, Faculty of Medicine and Health Sciences, University of Antwerp, Belgium*

*^4^Department of Infectious Disease Epidemiology, MRC Centre for Global Infectious Disease Analysis (MRC GIDA), and London Centre for Neglected Tropical Disease Research, School of Public Health, Imperial College London, London, UK*

*^5^STU Clinic, Sunyani Technical University, Sunyani, Bono Region, Ghana*

*^6^Deo Gratias Medical Laboratories, Sunyani, Bono Region, Ghana*

*^7^Kintampo Health Research Centre, Kintampo, Bono Region, Ghana*

*^8^Ghana Health Service, Regional Neglected Tropical Diseases (RNTD) Office, Regional Health Directorate, Sunyani, Bono Region, Ghana*

*^9^Amsterdam University Medical Centres, Academic Medical Centre at the University of Amsterdam, Department of Medical Microbiology, Experimental Parasitology Unit, Amsterdam, The Netherlands*

* **Corresponding author:** Kenneth Bentum Otabil, E**-**mail: [***Kenneth.otabil@uenr.edu.gh***](mailto:Kenneth.otabil@uenr.edu.gh)

**Instrument 1 Adherence to ivermectin treatment in onchocerciasis-endemic villages**

DATE: _____ / _____ / ________

FULL NAME OF INTERVIEWER: ________________________________________

DISTRICT _________________________________________

VILLAGE: _________________________________________

**Category 1: Demographic characteristics of study participants**

**Gender**

Male [ ] Female [ ]

**Age (years), specify ____**

**Current marital status**

Single [ ] Married [ ] Divorced [ ]. Widow/widower [ ]

**Education**

[ ] No formal education

[ ] Basic

[ ] Junior High/Secondary School

[ ] Senior High/Secondary School

[ ] Tertiary

**Religion**

Muslim [ ]. Christian [ ]. Traditional [ ] Others [ ]

**Ethnicity**

[ ] Indigenous of village [ ] Minority in village

**Occupation**

[ ] Farmer/Fishing/hunting [ ] Non-Farmers/fishers/hunter

**Duration of residence/stay in village**

Born and resided in the village [ ], not born in the village but resided in the village: < 1 year [ ] 1–3 years [ ] 4–7 years [ ] 8–11 years [ ] Other duration [ ], specify

**Category 2: Adherence to ivermectin**

1. Have you ever taken ivermectin before? Yes [ ] No [ ]
2. If No, what are the reasons? Fear of side-effects [ ], onchocerciasis not important [ ], severe disease (e.g. epilepsy) [ ], others [ ] , specify
3. If Yes, when was the first time you took ivermectin? (Year) ……….
4. When was the last time you took ivermectin? (Year) ………..
5. Do you systematically take ivermectin during each treatment round? Yes [ ] No [ ]
6. If Yes, how many times? One [ ] Two [ ] Three [ ] Four [ ] Five [ ] > 5 times [ ] Can’t remember [ ] Others [ ], specify
7. Did you take ivermectin during the last treatment round? Yes [ ] No [ ]
8. If No, why did you not take the drug?

Refused [ ] , if refused why? _____

Absent [ ] , if absent why? ______

Pregnant [ ]

Too young at last round [ ]

Previous side-effects [ ]

Alcohol intake [ ]

Distribution challenge [ ]: Treatment not available [ ] Treatment available but not reached by CDD [ ]

Onchocerciasis not important [ ]

Severe disease [ ], specify________.

Other reason [ ], specify________.

**Category 3: Perceptual factors and ivermectin adherence**

1. I believe that ivermectin intake is beneficial Yes [ ] No[ ]
2. Ivermectin intake is problematic Yes [ ] No [ ]
3. What do you know about onchocerciasis? (Tick as many as apply) A disease transmitted by bites of blackflies [ ], Causes skin disease [ ] Causes eye disease [ ] It is a spiritual disease [ ] Don’t know anything about the disease [ ]
4. What is the best medicine for onchocerciasis? Ivermectin [ ] Traditional medicine/Other [ ], specify
5. Is onchocerciasis a serious disease? Yes [ ] No [ ]
6. Why do you take ivermectin? (Tick as many as apply)

Someone encourages me to [ ]

Most people take it [ ]

For my own health [ ]

For the health of the community [ ]

Others [ ], specify……….

**Instrument 2 Moderator Guide for Focus Group Discussions and Interviews**

**Probe Questions:**

1. What are the different treatments you know for onchocerciasis (River blindness)?
2. Which of these do you think is the best? Explain why
3. How familiar are you with the yearly/twice yearly Mass Drug Administration of ivermectin?

**Follow-Up Questions:**

1. Have you ever taken ivermectin since they started distributing it in this village? If No, why? If Yes, what motivated you to take ivermectin the first time?
2. Do you think taking ivermectin annually/twice annually for several years has benefits? If No, why do you think this is the case? If Yes, can you explain these benefits?
3. Have you ever missed a round of the drug distribution? If Yes, Why did you miss it?
4. What do you like about the drug? and what you do not like about the drug?
5. What influences whether you take the drug?
6. What influences whether your friends take the drug?

**Exit Question:**

- Is there anything else you would like to say about the administration of ivermectin in your community?

| **Community** | **Population size^a^**  **[as per coverage records]** | **No. of CDDs** | **Gender of CDD** | **Ratio of CDDs to Population [as per coverage records]** |
| --- | --- | --- | --- | --- |
|  | **A. Tain District** | | | |
| Abekwai 2 | 150 [476] | 1 | Male | 1:150 [1:476] |
| Abekwai 3 | 700 [971] | 1 | Male | 1:820* [1:1,091] |
| Attakrom | 800 [520] | 1 | Male | 1:800 [1:520] |
| Kokomba | 120 [495] | 1 (shared with Abekwai 3) | Male | 1:820* [1:1,091] |
| **TOTAL** | 1,770 [2,462] | 3 (distinct CDDs) | Male | 1:590 [1:820] |
|  |  |  |  |  |
|  | **B. Wenchi Municipality** | | | |
| Adamukuraa | 80 [NA] | 1 (shared with 2 other communities) | Male | 1:>80* |
| Branam | 1,023 [NA] | 1 (shared with Gyabaa, State Farms, Wawama) | Male | 1:>1,023* |
| Gyabaa | 142 [NA] | 1 (shared with Branam) | Male | 1:1,165* |
| State Farms | 107 [NA] | 1 (shared with Branam) | Male | 1:1,130* |
| Wawama | 160 [NA] | 1 (shared with Branam) | Male | 1:1,183* |
| Kwanware | 150 [96] | 1 | Male | 1:150 [1:96] |
| Ottukrom | 109 [NA] | 1 | Male | 1:109 |
| Subinso 1 | 1,500 [1,080] | 1 | Male | 1:1500 [1:1,080] |
| Subinso 2 | 5,500 [2,451] | 2 | Males | 1:2,750 [1:1,225] |
| **TOTAL** | 8,771 [NA] | 7 (distinct CDDs) |  | 1:1,253 |
|  |  |  |  |  |

**Table S1** Information on community drug distributors (CDDs) in the study population

^a^ Population sizes were estimated at the time of the study but differ from the population sizes reported in Reference [25] of the Main Text, which were based on the CDD coverage records (and which can be substantially larger in some communities; given in square brackets where available). This means that the ratio of CDDs to population may be less favorable than that reported here. * Shared CDD.

**Table S2** Perceptual reasons affecting adherence to ivermectin for the 510 study participants.

For each question, the number of respondents is given in brackets

| **Variables (no. of respondents)** | **Overall (n=510)** |
| --- | --- |
| **Is onchocerciasis a serious disease?** (n=510) |  |
| No | 47 (9.2%) |
| Yes | 463 (90.8%) |
| **What is the best medicine for onchocerciasis?** (n=462) |  |
| Ivermectin | 429 (92.9%) |
| Traditional medicine/other | 33 (7.1%) |
| **I believe that ivermectin intake is beneficial (n=510)** |  |
| No | 53 (10.4%) |
| Yes | 457 (89.6%) |
| **Ivermectin intake is problematic^*^** (n=510) |  |
| No | 449 (88.0%) |
| Yes | 61 (12.0%) |
| **Why do you take ivermectin?**  (n=485) |  |
| For my own health | 388 (80.0%) |
| For the health of the community | 2 (0.4%) |
| Most people take it | 19 (3.9%) |
| Someone encourages me to take it | 70 (14.4%) |
| Other reasons | 6 (1.2%) |
| **If other reasons, specify (n=5)** |  |
| Because the government provides it | 1 (20.0%) |
| Do not know why I take the treatment | 1 (20.0%) |
| I was forced to take it | 3 (60.0%) |

* This question was quite broad and was used to find out if participants had any issue whatsoever with the mass drug administration (MDA) of ivermectin in general, including administration, distribution, adverse effects, etc. The question was explored in more detail in the FGDs and in-depth interviews.
